# TatD DNases Contribute to Biofilm Formation and Virulence in *Trueperella pyogenes*

**DOI:** 10.3389/fmicb.2021.758465

**Published:** 2021-11-15

**Authors:** Zehui Zhang, Yinfeng Liang, Lihui Yu, Menghan Chen, Yuru Guo, Zhiruo Kang, Chenghu Qu, Chunlian Tian, Dexian Zhang, Mingchun Liu

**Affiliations:** Key Laboratory of Livestock Infectious Diseases in Northeast China, Ministry of Education, College of Animal Science and Veterinary Medicine, Shenyang Agricultural University, Shenyang, China

**Keywords:** *Trueperella pyogenes*, TatD DNases, DNA hydrolysis, biofilm, virulence

## Abstract

TatD DNases are conserved proteins in a variety of organisms and are considered potential virulence factors in *Plasmodium falciparum* and *Streptococcus pneumoniae*. However, the function of TatD DNases has not yet been determined in *Trueperella pyogenes*, which causes various infections in animals and leads to economic losses. In this study, we describe the roles of TatD DNases in *T. pyogenes* (TpTatDs). A bioinformatics analysis was performed to investigate the sequence characteristics of TpTatDs, and then the ability of recombinant TatD proteins to hydrolyze DNA was determined in the presence of divalent cations. Moreover, we constructed *tatD*-deficient mutants. The biofilms formed by the wild-type and mutant strains were observed under a microscope. The mortality and bacterial load in the spleen of mice infected with the wild-type strain and *tatD*-deficient mutants were determined to obtain insights into the role of TatDs in the virulence of *T. pyogenes.* Two TatD DNases were identified in *T. pyogenes*. They were Mg^2+^-dependent DNases and exhibited DNA endonuclease activity. Compared with those formed by the parental strain, biofilms formed by mutants showed a significantly reduced thickness and biomass. Moreover, mutants produced a lower bacterial load in the spleen of mice and compromised virulence. Our data indicated that TatD DNases in *T. pyogenes* are involved in biofilm formation and required for virulence during infections.

## Introduction

*Trueperella pyogenes* (*T. pyogenes*) is a gram-positive, non-motile, and short rod-shaped organism characterized by hemolytic activity on blood agar (Rzewuska et al., 2019). It was formerly classified in the *Actinomycetaceae* family and was known as *Arcanobacterium pyogenes* but was reclassified as *T. pyogenes* according to the *16S rRNA* sequence (Yassin et al., 2011). Normally, *T. pyogenes* is found on the skin and mucous membranes of urogenital tracts, the upper respiratory tract, and udders of healthy animals and is known as an opportunistic pathogen that leads to a variety of infections in animals, including pneumonia, liver abscessation, mastitis, metritis, endocarditis, and osteoarthritis (Galán-Relaño et al., 2020). Infections caused by *T. pyogenes* often lead to substantial economic losses in livestock industries, and an understanding of how *T. pyogenes* leads to infection is crucial to reduce these losses.

A few virulence factors are proposed to be involved in infections caused by *T. pyogenes*. The hemolytic exotoxin pyolysin (PLO) lyses red blood cells from mammalian species and contributes to β-hemolysis when *T. pyogenes* is grown on blood-containing agar plates (Jost et al., 1999). Several virulence factors are involved in promoting adhesion, including fimbriae, extracellular matrix-binding proteins, and neuraminidases. Fimbriae, including FimA, FimB, FimC, FimE, and FimG, contribute to cell adhesion and the colonization of host tissue, and they are postulated to play equal roles in both commensal bacteria and the pathogen *T. pyogenes* (Liu et al., 2018). Moreover, *T. pyogenes* also expresses collagen-binding protein A (CbpA) to promote adhesion and enhance colonization by binding to extracellular matrix compounds on the surface of mammalian cells (Pietrocola et al., 2007). Two neuraminidases with the capability of cleaving the terminal sialic acid residues to expose the cryptic host cell receptors have been identified in *T. pyogenes* and promote tissue colonization by reducing mucus viscosity (Jost et al., 2001, 2002).

TatD DNases are synthesized in various organisms and are presumed to be involved in DNA repair, neutrophil extracellular trap (NET) hydrolysis and programmed apoptosis. For example, TatD DNases from *Escherichia coli* are capable of repairing DNA and are intimately linked to the Tat export pathway (Wexler et al., 2000; Matos et al., 2009; Chen et al., 2014). In addition, TatD DNases from *Plasmodium falciparum*, *African trypanosomes*, and *Streptococcus pneumoniae* degrade NETs to facilitate their escape from neutrophil immunity (Chang et al., 2016; Jhelum et al., 2018; Zhang et al., 2021). Moreover, TatD from *Saccharomyces cerevisiae* is involved in programmed cell death because of its nuclease activity (Qiu et al., 2005); similarly, TatD is crucial for apoptotic DNA degradation in *Caenorhabditis elegans*, *Leishmania* spp., and *Trypanosoma brucei* (Parrish and Xue, 2003; BoseDasgupta et al., 2008; Gannavaram and Debrabant, 2012).

Biofilms contribute to a variety of infections and are a community of microbial cells embedded in a matrix composed of extracellular polymeric substances (EPS) (Flemming et al., 2016; Petrova and Sauer, 2016; Sharma et al., 2019; Karygianni et al., 2020). Biofilm formation is a virulence characteristic for *T. pyogenes* invading host cells (Jost and Billington, 2005; Rzewuska et al., 2019; Meili, 2020). This study is anticipated to further reveal the potential roles of TatD DNases in the formation of biofilms by *T. pyogenes*.

## Materials and Methods

### Ethics Statement

Female Kunming mice (6–8 weeks of age) were purchased from Changsheng Biological Technology Co., Ltd. (Shenyang, China, Permit No.: SCXK (Liao) 2020-0001) and maintained under specific pathogen-free conditions for mouse survival experiments. The mouse livers were used to extract genomic DNA, and every effort was made to minimize suffering. Female New Zealand white rabbits (3 months of age) were purchased from Kangda Biological Technology Co., Ltd. (Qingdao, China, Permit No.: SCXK (Lu) 2020-0002) and raised under pathogen-free conditions for immunization. All animal procedures performed in the present study were conducted according to the animal husbandry guidelines of Shenyang Agricultural University. The Ethics Committee of Shenyang Agricultural University approved the laboratory animal experiments [Permit No.: SYXK (Liao) 2020-0001].

### *Trueperella pyogenes* Strains and Culture

*T. pyogenes* strain ATCC19411 was obtained from the American Type Culture Collection (Manassas, United States). *T. pyogenes* isolates (*n* = 19) were collected from dairy cows in Inner Mongolia and Liaoning, China and identified by *16S rRNA* gene sequencing (Zhang et al., 2017; Guo et al., 2020). *T. pyogenes* strains were cultured on Mueller-Hinton agar (MHA; Solarbio, Beijing, China) containing 5% (v/v) defibrinated sheep blood (Hopebio, Qingdao, China) in an incubator (5% CO_2_; Thermo Fisher, Shanghai, China) at 37°C for 36 h, and the colonies were inoculated in brain heart infusion (BHI) medium (Solarbio, Beijing, China) supplemented with 8% (v/v) fetal bovine serum (FBS; Gibco, Grand Island, United States). *E. coli* strains BL21 (DE3) (TransGen Biotech, Beijing, China) and Trans-1-T1 (TransGen Biotech, Beijing, China) were cultured in Luria-Bertani (LB) broth (Solarbio, Beijing, China) in a shaker at 200 rpm or on LB agar plates supplemented with kanamycin (50 μg/mL; Sigma, Shanghai, China) at 37°C.

### Bioinformatics Analysis of TpTatDs

*T. pyogenes* BMH-06-3 showed multidrug resistance; therefore, it was sequenced by Novogene Co., Ltd. (Beijing, China). The sequencing results indicated that it carried a variety of virulence in the genomic DNA (data not shown). The putative gene encoding TatD was retrieved from GenBank (accession No.: CP007003) by searching for annotations and matching with the genome of *T. pyogenes* BMH06-3. The physicochemical properties of TpTatDs were estimated using the ProtParam tool.^1^ We searched the *T. pyogenes tatD* genes in the Pfam protein family database^2^ to understand their domains. The SignalP 4.1 server^3^ was used to detect the presence of signal peptides in TpTatDs. TatD amino acid sequences from *Staphylococcus aureus*, *Bacillus anthracis*, *S. pneumoniae*, *E. coli*, and *T. pyogenes* were aligned using Clustal W^4^ and presented with ESPript 3.0.^5^ DNAMAN software was used to determine the identity rate for multiple sequence alignment of TatD DNase amino acid sequences. Structure models of TatD DNase 960 and TatD DNase 825 were built using SWISS-MODEL.^6^ The crystal structure of TatD DNase from the Protein Data Bank (PDB) was identified as a template for TpTatDs. The best models were selected based on a higher GMQE (confidence interval: 0–1) value and a QMEAN value (confidence interval: −4–0) closer to 0. The single best model was validated with the PROCHECK software and drawn using PyMOL. The crucial functional residues were identified using the 3DLigandSite prediction server^7^ with similar structures.

### The Distribution of *tatD* Genes

The distributions of *tatD960* and *tatD825* were determined among ATCC19411 and 19 *T. pyogenes* isolates using polymerase chain reaction (PCR). PCR amplifications were carried out in a 25 μL reaction mixture containing 0.1 μM each primer, 12.5 μL of PrimeSTAR HS (Premix) (2×; TaKaRa, Dalian, China), and 50 ng of template DNA. The primers (*tatD960*-F/R and *tatD825*-F/R) used to amplify *tatD* genes are listed in Supplementary Table 1. The PCR protocol was identical for all primers: 30 cycles of 10 s at 98°C, 5 s at 68°C, and 10 s at 72°C. The amplified DNA products were confirmed by 1.0% (w/v) agarose (Sigma, Shanghai, China) gel electrophoresis, and the bands were visualized with a gel imaging system (Azure Biosystems c300, United States). The amplicons were sequenced by Sangon Biotech (Shanghai, China). The phylogenetic tree of TpTatDs was constructed with MEGA5 software using the neighbor-joining method (Tamura et al., 2011). MEGA5 software was also used to infer a phylogenetic tree of 88 TatD DNases, including TatD DNases from bacteria, fungi, and protists. Sources and GenBank IDs of the 88 TatD DNases are shown in Supplementary Table 2. The phylogenetic trees were annotated using iTOL.^8^

### Expression and Purification of His-Tagged TatD Proteins

Sequences encoding *T. pyogenes TatD* 960 and *TatD* 825 were amplified from BMH06-3 genomic DNA using PrimeSTAR HS (Premix) (2×; TaKaRa). PCR amplification was carried out in a 50 μL reaction mixture containing each primer (0.1 μM, His-*tatD960*-F/R, or His-*tatD825*-F/R), 25 μL of PrimeSTAR HS (Premix) (2×; TaKaRa), and 100 ng of template DNA. The thermal cycling conditions were identical for primers: 30 cycles of 10 s at 98°C, 5 s at 68°C, and 10 s at 72°C. pET28a-*tatD960* was constructed using the restriction enzymes *Nde*I and *Hin*dIII (TaKaRa, Dalian, China), and the *tatD960* amplicon was cloned into the digested expression vector pET28a (Supplementary Figure 1A). The same approach was used to construct pET28a-*tatD825* with the restriction enzymes *Bam*HI and *Hin*dIII (TaKaRa, Dalian, China) (Supplementary Figure 1B). The recombinant vectors were transformed into the *E. coli* strain Trans-1-T1 (TransGen Biotech, Beijing, China). The recombinant vectors were identified by PCR, restriction enzyme analysis, and nucleotide sequencing. The recombinant vectors were transformed into *E. coli* strain BL21 (DE3) (TransGen Biotech) for expression. His-tagged recombinant TatD proteins were expressed in *E. coli*. The soluble proteins were purified using a His GraviTrap affinity chromatography column (GE Healthcare, Shanghai, Sweden). The purified protein was evaluated using sodium dodecyl-sulfate polyacrylamide gel electrophoresis (SDS–PAGE) and Western blot analyses. Briefly, the recombinant protein was electrophoresed on 12% SDS–PAGE gels (Solarbio, Beijing, China) and then transferred to a 0.45 μm polyvinylidene fluoride (PVDF) membrane (Millipore, Shanghai, China) using the wet transfer method. Rabbit polyclonal anti-6 × His IgG (1:2,000) (BBI Life Science, Shanghai, China) was used as the primary antibody, and alkaline phosphatase (AP)-labeled goat anti-rabbit IgG (1:5,000) (BBI Life Science) was used as the secondary antibody. PVDF membranes (Millipore, Shanghai, China) were incubated with BCIP/NBT solution (Solarbio, Beijing, China) for 5 min, and images were acquired.

### Analysis of the DNase Activity of TpTatDs

Linear DNA was extracted from mouse livers using a TIANamp Genomic DNA Kit (TIANGEN Biotech, Beijing, China) according to the manufacturer’s protocol. The pBR322 plasmid (1 μg/mL) was purchased from TaKaRa Biomedical Technology Co., Ltd. (Dalian, China). The concentration of TpTatDs was quantified using a bicinchoninic acid (BCA) Protein Assay Kit (Abcam, Cambridge, United Kingdom). Linear DNA was incubated with TpTatDs (final concentrations: 0.5, 1, 2, 3, 4, and 5 μM) in phosphate-buffered saline (PBS; pH = 7.4; Solarbio, Beijing, China) in a final volume of 20 μL at 37°C, and reaction times were 5, 15, 30, 45, and 60 min. PBS (pH = 7.4, Solarbio) was used as a negative control. Six divalent metal ions were added to the reactions to test the facilitation of divalent metal ions. The plasmid pBR322 (TaKaRa) was mixed with TatDs, and the solution was incubated at 37°C for 1 h to investigate the effect of TatD on circular DNA. The cultured mixture was analyzed using 1.0% agarose gel electrophoresis to determine whether the circular DNA was hydrolyzed. The DNase activity of TpTatDs was quantitatively analyzed by measuring the fluorescence intensity of PicoGreen reagent (Invitrogen, United States). The assay is based on the ability of PicoGreen reagent to bind to double-stranded DNA and increase its fluorescence. The DNA substrate (200 ng) was incubated with TpTatDs (2 μM) at different temperatures or pH values for 20 min. PicoGreen diluted 200-fold using Tris-ethylenediaminetetraacetic acid (EDTA; TE) buffer (pH = 7.5; Solarbio, Beijing, China) was used as the working reagent. The mixture of the DNA substrate and TpTatDs was incubated with 100 μL of PicoGreen working reagent at 25°C for 5 min. Two hundred microliter reaction mixtures were added to a 96-well fluorescence microtiter plate, and the fluorescence of the samples was measured using a VICTOR Nivo Multimode Plate Reader (PerkinElmer, Waltham, United States) at excitation and emission wavelengths of 480 and 520 nm, respectively. The activity of TatD DNase was calculated using the following equation: DNase activity (%) = [(fluorescence of the DNA control—fluorescence of the treated sample)/fluorescence of the DNA control] × 100 (Jhelum et al., 2018).

### Preparation of Anti-TpTatD Polyclonal Antibodies

The anti-TpTatD 960 polyclonal antibody and anti-TpTatD 825 polyclonal antibody were prepared by immunizing female New Zealand white rabbits with a total of 400 μg/rabbit His-labeled recombinant protein emulsified in complete Freund’s adjuvant (Sigma, Shanghai, China) and incomplete Freund’s adjuvant (Sigma, Shanghai, China). The sequences of *tatD960* and *tatD825* were optimized for expression in *E. coli* by cloning into the pGEX4T-1 vectors, and glutathione S-transferase (GST)-tagged recombinant proteins were expressed in *E. coli* and purified using glutathione Sepharose affinity chromatography (GE Healthcare, Shanghai, Sweden). GST-tagged recombinant proteins were used to test the antibody titer. When the antibody titer reached 1:16,000, the immune sera were collected and stored at −80°C until use.

### Detection of TatD DNases in Bacterial Cells and Extracellular Supernatant

One milliliter of an overnight culture of *T. pyogenes* BMH06-3 was added to 100 mL of BHI medium (Solarbio) containing 8% FBS (Gibco, Grand Island, United States). The culture supernatant was collected by centrifugation and lyophilized using a vacuum freeze dryer (Haozhuang Instrument Co., Ltd., Shanghai, China) as described by Zhang et al. (2021). In addition, the bacterial cells were mixed with 2 mL of bacterial protein extraction reagent (Sigma, Shanghai, China) and 20 μL of phenylmethanesulfonylfluoride (PMSF; Sigma, Shanghai, China). The suspension was sonicated with a Sonics Vibra-Cell Ultrasonic Processor (VCX-150, Artisan Technology Group, Newtown, United States), and total proteins from bacterial cells were collected by centrifugation as previously described (Guo et al., 2020). Total bacterial proteins and the extracellular supernatant were probed using Western blotting. Samples were separated on 12% SDS–PAGE gels and transferred to PVDF membranes (Bio-Rad, United States). Membranes were blocked with 5% (w/v) bovine serum albumin (BSA) in tris-buffered saline with Tween 20 (TBST) for 12 h at 4°C, and rabbit immune serum (1:2,000) was used as the primary antibody and incubated with the membrane for 12 h at 4°C. IgG (1:20,000; ZSGB-BIO, Beijing, China) was incubated with the membrane for 1 h at 37°C. AP-labeled goat anti-rabbit IgG (1:5,000) (BBI Life Science) was used as the secondary antibody. PVDF membranes (Millipore, China) were incubated with 5-bromo-4-chloro-3-indolyl-phosphate (BCIP)/nitro blue tetrazolium (NBT) solution (Solarbio, Beijing, China), and the target protein was visualized.

### Construction of *tatD*-Deficient Mutants

**FIGURE 1:**
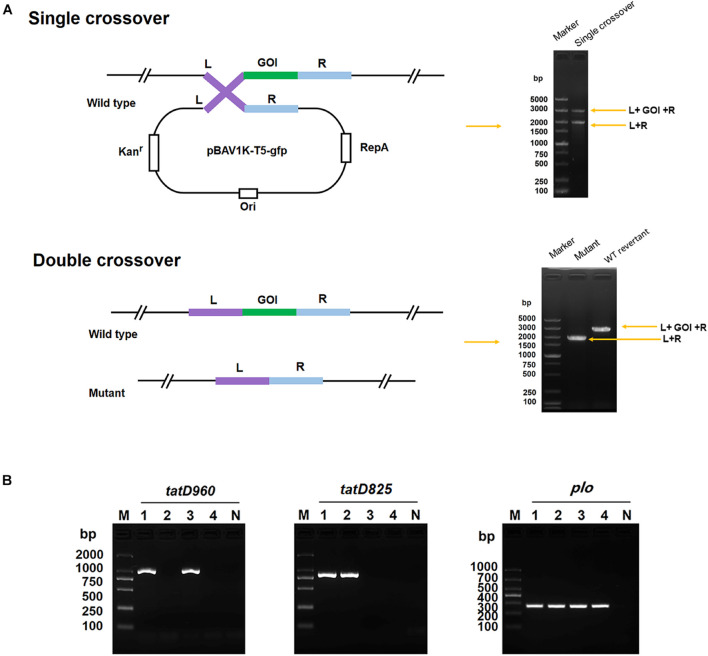
Construction of the *tatD*-deficient mutants in *T. pyogenes* strain BMH06-3 based on the plasmid PBAV1K-T5-gfp. **(A)** Illustration of markerless knockout construction in *T. pyogenes*. Kan, kanamycin; L, upstream homologous arm; R, downstream homologous arm; GOI, gene of interest; and WT, wild-type. **(B)** Identification of *tatD*-deficient strains using PCR and sequencing. The mutants were identified by PCR using three primer pairs: *tatD960* F/R, *tatD825* F/R, and *plo* F/R. Lane 1, BMH06-3; Lane 2, BMH06-3Δ*tatD960*; Lane 3, BMH06-3Δ*tatD825*; Lane 4, BMH06-3Δ*tatD960*Δ*tatD825*. M, DNA Marker DL2000/DL1000.

Mutants of *T. pyogenes* strain BMH06–3 deficient in *tatD960*, *tatD825*, and both *tatD960* and *tatD825* (double deficiencies) were constructed. The process of knockout based on pBAV1K-T5-gfp, which was described by Bryksin and Matsumura (2010), was performed using double-crossover mechanisms. The enzyme cleavage sites selected for this study were marked on pBAV1K-T5-gfp and are shown in Supplementary Figure 2. The primers used to construct the *tatD*-deficient mutants are listed in Supplementary Table 1. Briefly, the 1016 bp upstream sequence and 947 bp downstream sequence of *tatD960* (including restriction enzyme digestion sites and protective bases) was amplified. The two PCR products (containing a *Sal*I site) were ligated using T4 ligase. The fused fragment was purified and digested with *Bam*HI and *Spe*I (TaKaRa, Dalian, China). Then, the fragment was cloned into pBAV1K-T5-gfp, which had been digested with the same enzymes, to generate pBAV1K-T5-gfp:*tatD960* up-down. The plasmid pBAV1K-T5-gfp:*tatD825* up-down was constructed using the same method with slight modification. The 1,022 bp upstream sequence and 1,023 bp downstream sequence of *tatD825* (including restriction enzyme digestion site, protective bases, and overlap length) were ligated using overlapping PCR, and the fused fragment was digested with *Spe*I and *Apa*I (TaKaRa, Dalian, China) and cloned into pBAV1K-T5-gfp. *T. pyogenes* was transformed with the recombinant construct by electroporation, as described previously (Zhang et al., 2019). The strategy used for obtaining and confirming the mutants is shown in Figure 1 (Cripps et al., 2009). As BMH06-3 is susceptible to kanamycin (4 μg/mL) and the recombinant construct containing a kanamycin resistance gene, transformants were selected on MHA plates (Solarbio) containing 5% (v/v) defibrinated sheep blood (Hopebio) and 30 μg/mL kanamycin (Sigma, Shanghai, China). The formation of single crossovers was confirmed by PCR using genomic DNA and primers *tatD960* L-F/R-R and *tatD825* L-F/R-R. Single crossovers were subcultured repeatedly (5 subcultures) without kanamycin in BHI medium at 37°C for 12 h and cultured on a non-selective plate. The resulting colonies were screened for kanamycin resistance by replica plating to kanamycin selection plates (30 μg/mL kanamycin) and non-selective plates. Presumptive mutants were distinguished from single crossovers by their sensitivity to kanamycin (30 μg/mL). Clones suspected to be mutants were verified by PCR and nucleotide sequencing. *T. pyogenes* strain BMH06-3Δ*tatD960*Δ*tatD825* (double deficiencies) was generated by transforming pBAV1K-T5-gfp:*tatD960* up-down into strain BMH06-3Δ*tatD825*. The mutants were confirmed by colony PCR and nucleotide sequencing.

### Determination of the Growth Curve

We evaluated the effect of *tatD* gene deletion on the growth rate of *T. pyogenes* by measuring growth curves. One milliliter of the wild-type strain or *tatD*-deficient mutants was cultured to the logarithmic growth phase (1 × 10^8^ colony-forming unit (CFU)/mL) and added to BHI medium containing 8% FBS. Bacterial suspensions were incubated at 37°C at a speed of 150 rpm, and the OD was measured at 600 nm each hour. After incubation for 40 h, the bacterial growth curve was plotted and analyzed.

### Biofilm Quantification and Observation

An inoculum of *T. pyogenes* (1 × 10^6^ CFU/mL) was added to 24-well plates (Corning, United States) and glass bottom cell culture dishes (NEST, Wuxi, China) to measure the biomass of biofilms at different time points using the method described by Zhao et al. (2013). The bacterial suspension was incubated under static conditions for 2, 6, 12, 24, 48, and 72 h. The time point of biofilm maturation was determined by performing crystal violet (CV; Solarbio, Beijing, China) staining and microscopic observations.

*T. pyogenes* strain BMH06-3 and mutants were incubated under static conditions to form biofilms. Biofilm quantification was carried out as previously described (Matysik and Kline, 2019). One milliliter of the *T. pyogenes* culture (1 × 10^6^ CFU/mL) was added to each well of 24-well plates (Corning, Shanghai, China). Subsequently, the samples were treated with DNase I (TransGen Biotech; final concentrations: 0, 50, and 100 U/mL). The bacterial suspension was incubated at 37°C under static conditions. After static incubation, 24-well plates were washed gently with PBS (pH = 7.4, Solarbio) three times to remove planktonic cells. Biofilm populations were stained with 0.1% crystal violet (CV; Solarbio) for 15 min, and the excess CV was removed by washing with PBS (pH = 7.4, Solarbio). One milliliter of 95% ethanol (Sigma, Shanghai, China) was added to the wells to visualize the biofilms, and the optical density (OD) was measured with a multimode plate reader (VICTOR Nivo) at 595 nm.

Confocal laser scanning microscopy (CLSM) and scanning electron microscopy (SEM) were used to observe biofilms formed by the wild-type and mutant strains. The bacterial suspension was incubated in glass bottom cell culture dishes (NEST). Biofilms in cell culture dishes were washed in PBS (pH = 7.4, Solarbio) three times to remove the planktonic cells, stained with the LIVE/DEAD BacLight Bacterial Viability Kit (Invitrogen, Camarillo, United States) according to the manufacturer’s protocol, and washed with PBS (pH = 7.4, Solarbio). Syto9 was used to stain DNA in all cells. Propidium iodide was used to stain the DNA in dead cells, as it cannot cross the membrane of live cells. Images were captured with a confocal microscope (Leica Camera AG, Solms, Germany). A suspension of *T. pyogenes* in 24-well plates was incubated on cell slides. All cell slides washed three times with 0.1 M PBS (pH = 7.4, Solarbio), fixed overnight at 4°C with 2.5% glutaraldehyde (Sigma, Shanghai, China), dehydrated in an ethanol gradient, and finally dried. After platinum coating, samples were imaged using an electron microscope (HITACHI, Tokyo, Japan).

### Relative eDNA Quantification

EPS were extracted from the biofilms of the wild-type and mutant strains using an ultrasonic method (DeFrancesco et al., 2017). Briefly, the medium was removed, and the remaining adherent cells were washed with PBS (pH = 7.4, Solarbio). The biofilm was resuspended in 1 mL of a 0.01 M KCl solution (Solarbio, Beijing, China), and the OD of each well was measured at 595 nm using a multimode plate reader (VICTOR Nivo) to quantify the biomass of the sample used for eDNA extraction. Next, a Sonics Vibra-Cell Ultrasonic Processor (Artisan Technology Group) was used to disperse the *T. pyogenes* cells, and the parameters were 45 W, 20 kHz, 6 cycles, run 5 s and pause 5 sec. The sonicated suspension was centrifuged at 5000 × *g* for 10 min at 4°C, and the cells were then removed from the supernatant by filtration through a 0.22 μm membrane filter (Millipore). The total eDNA content was determined using PicoGreen reagent as described above. The relative biomass of the biofilm in each well was calculated by setting the biomass of the wild-type strain to 1. Finally, the total eDNA quantified was normalized to the relative biomass of each sample to obtain the relative eDNA content of the biofilm (Bao et al., 2015).

### Triton 100-X Introduced Autolysis Assay

Determination of autolysis of *T. pyogenes* was performed according to Wang et al. (2019). *T. pyogenes* was grown in BHI medium containing 8% FBS at 37°C with shaking until the late stage of logarithmic growth. The cells were harvested through centrifugation and washed twice with 50 mM Tris–HCl buffer (pH = 7.5). *T. pyogenes* were resuspended in a buffer containing 50 mM Tris–HCl (pH 7.5) and 0.1% Triton X-100 and were subsequently incubated at 37°C with shaking. The OD_58__0n__*m*_ value in the wild-type strain and *tatD*-deficient mutants was determined every 0.5 h (the maximum time was 6 h). The amount of bacterial cell lysis was represented using the ratio of immediate absorbance to initial absorbance as a percentage.

### Mice Experiments

Kunming mice were selected for survival experiments since they show high disease resistance and adaptability. Ten 6- to 8-week-old female Kunming mice were selected and randomly divided into 5 groups (10 mice/group). Mice in Groups I, II, III, IV, and V were infected intraperitoneally with BMH-06-3, BMH-06-3Δ*tatD*960, BMH-06-3Δ*tatD*825, BMH-06-3Δ*tatD960*Δ*tatD825*, and 0.9% saline (Solarbio), respectively. Mice in Groups I-IV were injected with 1 × 10^9^ CFU per mouse. The mice were monitored for symptoms every 4 h for 14 days.

Mice were divided into four groups, and mice in Group I were injected intraperitoneally with the wild-type strain, mice in Group II were injected intraperitoneally with the *tatD960*-deficient strain, mice in Group III were injected intraperitoneally with the *tatD825*-deficient strain, and mice in Group IV were injected intraperitoneally with the *tatD*960 and *tatD*825 double deficient strain. Six mice in each group were injected intraperitoneally with the bacterial solution (1 × 10^8^ CFU). Mice injected intraperitoneally with 0.9% saline (Solarbio) were used as the control group. After 24 h, the *T. pyogenes* load was determined in the spleen of mice. Briefly, mice were euthanized by cervical dislocation, and spleens were harvested. The spleen was homogenized in 1 mL of PBS (pH = 7.4; Solarbio) using a tissue lyser (Jingxin Industrial Development Co., Ltd., Shanghai, China). The homogenates were serially diluted in sterile PBS (pH = 7.4; Solarbio) and plated on MHA plates containing 5% (v/v) sheep blood (Hopebio) and 0.1% colistin sulfate salt.

### Statistical Analysis

All assays were carried out in triplicate. The data are presented as the mean ± standard deviation (SD). Statistical analyses were performed using one-way of variance (ANOVA) with SPSS Statistics V17.0 and GraphPad Prism 5.0 software. For all analyses, statistical significance was defined as ^∗∗^*p* < 0.01 and ^∗∗∗^*p* < 0.001.

## Results

### Sequence Characteristics of TpTatDs

**FIGURE 2:**
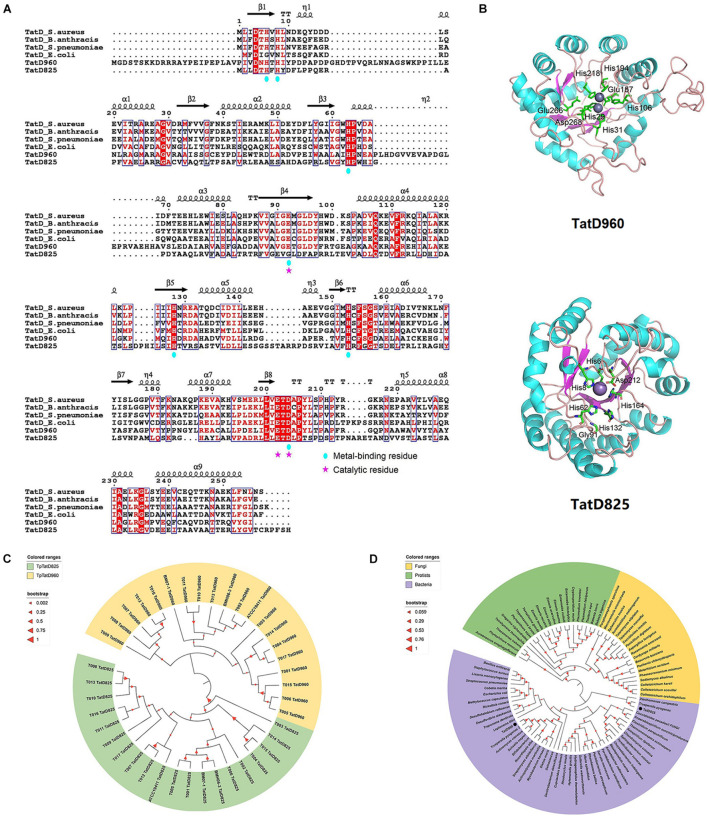
Sequence characteristics and phylogenetic relationships of TatD DNases in *T. pyogenes*. **(A)** Amino acid sequence alignment of TpTatDs using ESPript with its homologs of *S. aureus*, *S. pneumoniae*, *B. anthracis* and *E. coli*. The conserved residues are highlighted in white on a red background. Similar residues are presented in red in blue boxes. The residues contributing to metal binding and catalysis are marked with blue circles and pink stars, respectively. **(B)** Structural modeling of TpTatDs using SWISS-MODEL. The best match for TatD DNase 960 was found to be with *E. coli* TatD (PDB ID: 1YIX), and the best match for TatD DNase 825 was *E. coli* TatD (PDB ID: 3GG7). Metal-binding and catalytic residues in TpTatDs are highlighted in the drawing. **(C)** Phylogenetic tree of TatD DNase 960 (yellow) and TatD DNase 825 (green) in 20 *T. pyogenes*. **(D)** Phylogenetic tree of TatD DNases from bacteria (purple), fungi (yellow) and protists (green). TatD DNases of *T. pyogenes* are indicated with black circles.

Genes encoding two TatD DNases with similar structural domains were identified in the genomic DNA of *T. pyogenes* TP8 (accession No.: CP007003), and they are collectively referred to as *tatD960* (locus tag: *X956_RS01795*) and *tatD825* (locus tag: *X956_RS00220*) according to the length of their nucleotide sequences (Supplementary Figure 3). The predicted molecular weights of TatD DNase 960 and TatD DNase 825 were 34.7 and 30.0 kDa, respectively. A search of the Pfam protein family database showed that the proteins encoded by *tatD* genes belong to the TatD (PF01026) family, which is a family of proteins with TIM-barrel folds and conserved amino acid residues associated with DNase activity. Both TpTatDs lacked signal peptides. The amino acid sequences of TpTatDs were compared with those of homologs from *S. aureus*, *E. coli*, and *S. pneumoniae*, as shown in Figure 2A. The sequence identity of TatD DNase amino acid sequences from *S. aureus*, *B anthracis*, *S. pneumoniae*, *E. coli*, and *T. pyogenes* was 35.76%. The amino acid sequences of TatD DNase 960 and TatD DNase 825 from *T. pyogenes* established the possible three-dimensional structures of TatD DNases (Figure 2B). Based on the sequence analysis, we predicted that His29, His31, His106, Glu157, His194, His218, and Asp268 may be involved in binding metal ions in TatD DNase 960 and that Glu157, Glu266, and Asp268 may play key roles in the catalytic reaction. In TatD DNase 825, His6, His8, His62, Gly91, His132, His164, and Asp212 may be involved in binding metal ions, while Gly91 and Asp212 may be crucial for the catalytic reaction. The sequencing of PCR products showed that the detection rate of *tatD960* and *tatD825* in 20 *T. pyogenes* isolates was 100% (Supplementary Figure 4). The neighbor-joining tree of TatD DNases in *T. pyogenes* revealed that TatD DNase 960 and TatD DNase 825 belonged to different clusters and that the TatD DNase was highly evolutionarily conserved in the isolates (Figure 2C). We found that TatD DNases in bacteria exhibited similarity at the evolutionary level and were distinguished from fungi and protists (Figure 2D). The conserved sequences of TatD DNases from *T. pyogenes* and other bacteria indicated that they may have similar functions.

### TpTatDs Employ Divalent Metal Ions for Endonuclease Activity

**FIGURE 3:**
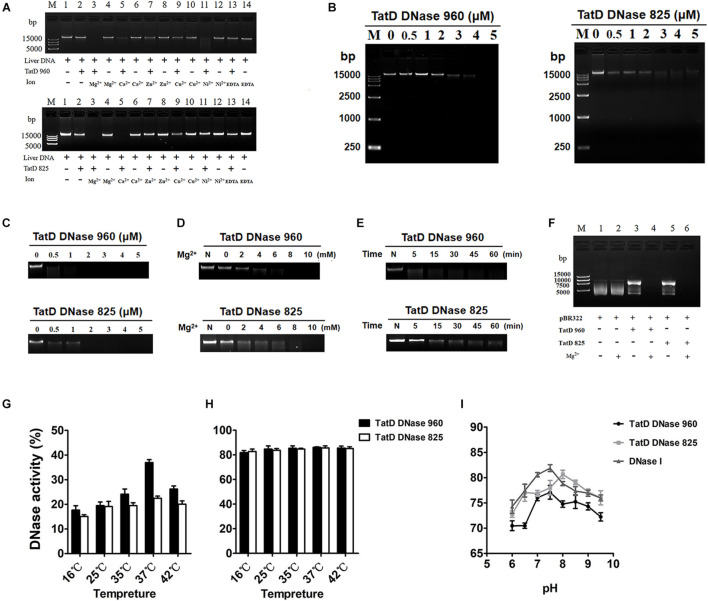
The DNase activity of TpTatDs. **(A)** The effects of divalent metal cations on the DNase activity of TpTatDs. Linear DNA was incubated in the absence (lanes 1, 4, 6, 8, 10, 12, and 14) or presence (lanes 2, 3, 5, 7, 9, 11, and 13) of TpTatDs and a variety of metal ions for 60 min at 37°C. DNA was visualized by agarose gel electrophoresis. The concentration of metal ions was 10 mM. **(B)** TpTatDs can weakly degrade linear DNA without metal cations. TpTatDs at various concentrations (0–5 μM) were incubated with linear DNA (200 ng) in PBS (pH = 7.4) at 37°C for 1 h. **(C,D)** Mg^2+^ can promote the hydrolysis of linear DNA by TpTatDs. TpTatDs (2 μM) with Mg^2+^ (10 mM) were able to completely hydrolyze DNA at 37°C for 1 h. **(E)** TatD DNase 960 (5 μM) was able to completely hydrolyze linear DNA within 1 h, and TatD DNase 825 (5 μM) hydrolyzed most of the linear DNA. **(F)** The ability of TpTatDs to digest plasmid DNA substrates. Two hundred nanograms of plasmid pBR322 was incubated with 2 μM TatD DNase 960/TatD DNase 825 at 37°C for 1 h. Mg^2+^ (10 mM) was added to the complex as a stimulator. Mg^2+^ can promote TatD DNases to hydrolyze pBR322. **(G,H)** Analysis of the effect of temperature on the DNase activity of TpTatDs in the absence or presence of Mg^2+^ by PicoGreen assay. **(I)** Analysis of the effect of pH on the DNase activity of TpTatDs in the presence of Mg^2+^ by PicoGreen assay. The reactions were performed in PBS with pH values ranging from 6 to 9.5. DNase I was used as a control.

The molecular weights of TatD DNase 960 and TatD DNase 825 with His tags were 37 kDa and 32 kDa, respectively (Supplementary Figure 5). An in-gel DNase activity assay showed that TatD DNase 960 and TatD DNase 825 have DNA degradation activity. Here, divalent metal ions, including Mg^2+^, Ca^2+^, and Ni^2+^, promoted the DNA hydrolysis activity of TatD DNase 960 (Figure 3A). The hydrolysis activity of TatD DNase 960 depended on Mg^2+^ (Figures 3B–D). Increasing the concentration of TatD DNase 960 or prolonging the reaction time improved the ability of TpTatD to hydrolyze DNA (Figures 3B,C,E). TatD DNase 960 also hydrolyzed circular DNA, and Mg^2+^ promoted the degradation of the plasmid pBR322 by TatD DNase 960 (Figure 3F). The effects of temperature and pH on the DNase activity of TatD DNase 960 were analyzed using a PicoGreen assay. The optimal reaction temperature for TatD DNase 960 was 37°C (Figures 3G,H). Recombinant TatD proteins were incubated with mouse liver DNA in PBS with a pH ranging from 6 to 9.5 to determine the pH dependence of DNase activity. The recombinant TatD 960 protein showed DNase activity over a wide pH range, with the maximum DNase activity observed at pH 8.0 (Figure 3I). Similar to TatD DNase 960, the catalytic activity of TatD DNase 825 showed metal ion dependence. Mg^2+^ and Ca^2+^ promoted DNA hydrolysis by TatD DNase 825 (Figure 3A). The DNase activity of TatD DNase 825 increased when concentrations increased and reaction time was prolonged (Figures 3B,C,E). Based on these results, TatD DNase 825 is also a Mg^2+^-dependent DNA endonuclease (Figures 3D,F). The optimum reaction temperature for TatD DNase 825 was 37°C, and the maximum activity of TatD DNase 825 was observed at pH 7.5 (Figures 3G–I). The reaction conditions and enzymatic activity of TpTatDs were similar to those of DNase I, showing industrial applicability (Figure 3I).

### TatD Proteins Are Expressed Intracellularly in *Trueperella pyogenes*

TpTatD960 and TpTatD825 were detected only in total bacterial cells, while they were not detected in culture supernatants. This indicated that TatD proteins may not be secreted from *T. pyogenes* under *in vitro* culture conditions (Supplementary Figure 6).

### Growth Rate of Wild-Type and *tatD*-Deficient Mutant Strains

No significant change was observed in the growth curves of wild-type and *tatD*-deficient mutant strains cultured under the same conditions. From 0 to 4 h, the bacteria grew slowly; at 4–16 h, the bacteria grew logarithmically and rapidly; and after 16 h, bacterial growth reached a plateau and was in equilibrium (Supplementary Figure 7).

### Quantification and Observations of *Trueperella pyogenes* Biofilms

**FIGURE 4:**
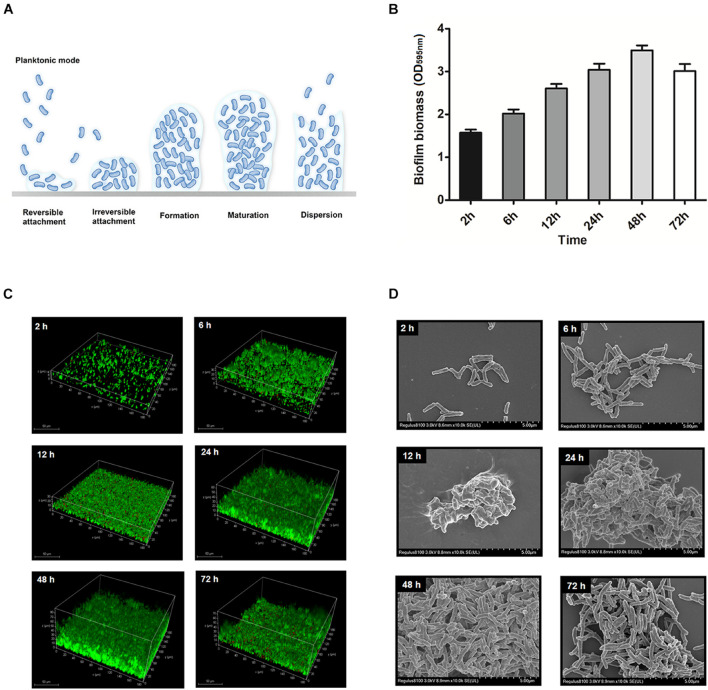
Model for static *T. pyogenes* biofilm development. **(A)** With respect to biofilm development, the 6 steps were planktonic stage, reversible attachment, irreversible attachment, biofilm formation, biofilm maturation and dispersion. **(B)** Biofilm biomass at different time points stained with CV. **(C)** BMH06-3 biofilms were grown on cell culture dishes stained with Syto9 (green) and propidium iodide (red). The volume projection of a Z-stack is shown. **(D)** Scanning electron micrographs of BMH06-3 biofilms at × 10,000 magnification.

The development of *T. pyogenes* biofilms is shown in Figure 4. Reversible attachment of planktonic bacteria occurred after *T. pyogenes* was incubated under static conditions for 2–6 h. Since the adhesion of planktonic bacteria was reversible at the beginning of the period, only a few cells underwent colonization. The irreversible colonization phase occurred within 12 h, and a significant increase in biomass was quantified and observed. After 24–48 h of static culture, the biofilm formed and developed to maturity. Lysis of the biofilm occurred at 72 h, when a reduction in biomass and free bacteria was observed. We determined that *T. pyogenes* tends to mature in static culture for 48 h and that its formation can be detected.

### Deficiencies in *tatD* Genes Decrease Biofilm Formation by *Trueperella pyogenes*

**FIGURE 5:**
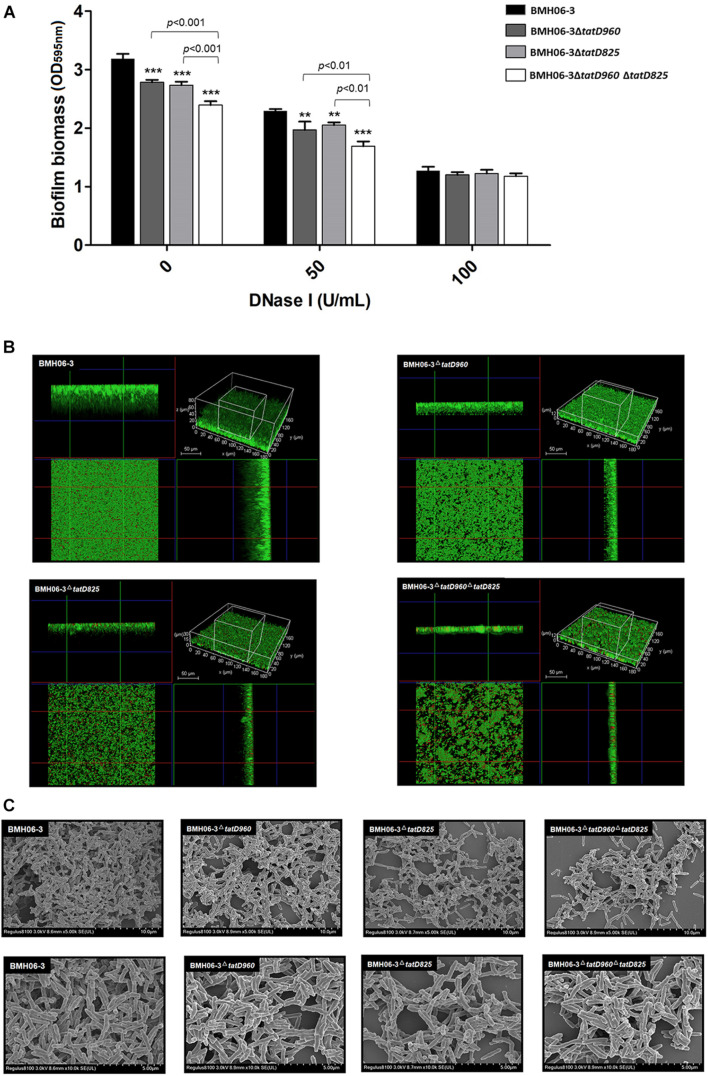
Biofilm biomass assay and morphology with the wild-type strain and *tatD*-deficient mutants. **(A)** eDNA is important for *T. pyogenes* BMH06-3 biofilm formation. The addition of DNase I (100 U/mL) at the time of inoculation eliminated the biofilm biomass difference among the wild-type strain and *tatD*-deficient mutants. Statistical analysis was carried out using one-way of variance (ANOVA) on SPSS Statistics V17.0. Statistical significance compared with the control group (BMH06-3) was defined as **p < 0.01 and ***p < 0.001. **(B)** CLSM analysis of biofilm thickness and denseness after 48 h of incubation in the static state. **(C)** SEM images of *T. pyogenes* biofilms after incubation for 48 h. Biofilms were imaged at × 5,000 (top) and × 10,000 (bottom) magnification.

Biofilm quantification results showed that after incubation without DNase I, the biofilm biomass of mutants, including BMH06-3Δ*tatD960*, BMH06-3Δ*tatD825*, and BMH06-3Δ*tatD960*Δ*tatD825*, was significantly (*p* < 0.001) lower than that of BMH06-3 (Figure 5A). DNase I induced a reduction in biofilm formation of *T. pyogenes*. There was no significant difference in biofilm biomass between the wild-type strain and *tatD*-deficient mutants treated with 100 U/mL DNase I, and all biofilm biomasses were reduced (Figure 5A). These data suggest that eDNA is important for biofilm formation. We analyzed the biofilm structure and viability, and the results indicated that mutants did not lose the ability to form biofilms; however, the biofilm biomass was effectively reduced. The biofilm biomass and the depth of mutants were reduced compared to the wild-type strain, as shown qualitatively in Figure 5B. The morphological features of biofilms formed by different strains were observed under SEM (Figure 5C). Deficiencies in the *tatD* genes affected the dense structure of biofilms, especially the double mutation of *tatD960* and *tatD825*, in which a tendency toward decreased biofilm formation was observed. These observations indicate that deficiencies in *tatD* genes may lead to reduced biofilm formation and an unstable biofilm structure in *T. pyogenes*.

### Relative eDNA Production and Autolytic Activity Are Reduced in the *tatD*-Deficient Mutants

**FIGURE 6:**
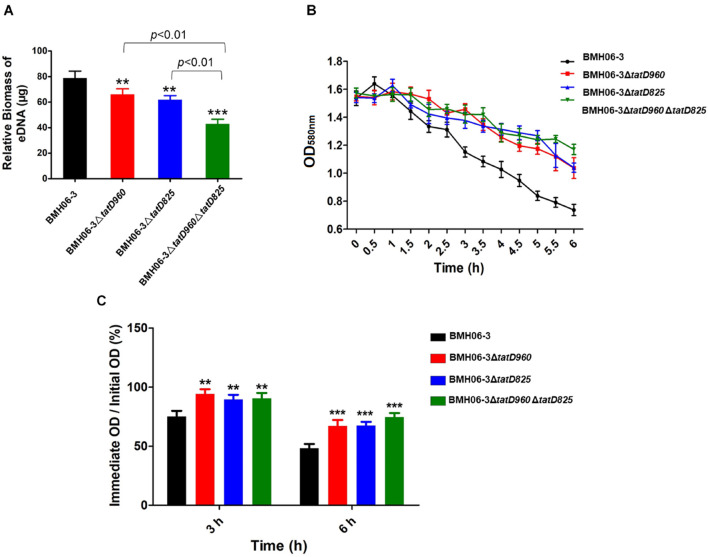
The reduction in biofilm formation in *tatD*-deficient mutants may be associated with the reduced autolysis and relative eDNA content in the biofilm. **(A)** The EPS of *T. pyogenes* biofilm were extracted. Relative eDNA in the EPS was quantified using the PicoGreen assay. **(B)** The autolytic ability of *T. pyogenes* was detected by treatment with 0.1% Triton X-100 and measuring the OD_58__0n__*m*_ value. **(C)** *tatD* deficiency reduces the autolytic ability of *T. pyogenes*. The wild-type strain BMH06-3 was used as a control group. Statistical analysis was carried out using one-way of variance (ANOVA) on SPSS Statistics V17.0. Statistical significance compared with the control group was defined as **p < 0.01 and ***p < 0.001.

After incubation for 48 h, the wild-type strain produced significantly higher levels of eDNA than the mutants, particularly BMH06-3Δ*tatD*960Δ*tatD*825 (Figure 6A). The *tatD*-deficient mutants showed a reduction in Triton X-100-induced autolysis compared with the wild-type strain (Figure 6B). The percentage of bacterial cells lysed at 3 h and 6 h is shown in Figure 6C. A significant reduction in autolysis was observed in the *tatD*-deficient mutants compared to the wild-type strain.

### *tatD* Deficiency Leads to Compromised Virulence

**FIGURE 7:**
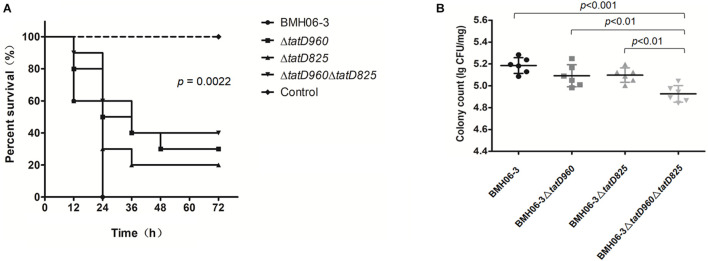
Deficiencies in *tatD* genes compromise *T. pyogenes* virulence in mice. **(A)** Kaplan-Meier survival curves of mice infected intraperitoneally with the *T. pyogenes* wild-type strain or *tatD*-deficient mutants (10 mice/group). The log-rank test was used to compute the *p*-value in the Kaplan-Meier survival analysis. **(B)** Mice infected intraperitoneally with BMH06-3- or *tatD*-deficient mutants in BMH06-3 (6 mice/group) were assessed for *T. pyogenes* load in the spleen at the 24 h time point. The spleen was processed for colony counting by the dilution plating method. The bar represents mean of triplicates.

All the mice in Group I that were injected with BMH06-3 (10^9^ cfu per mouse) succumbed in 24 h. Three groups showed higher survival rates than Group I: 30% for Group II injected with BMH06-3Δ*tatD960*, 20% for Group III injected with BMH06-3Δ*tatD825*, and 40% for Group IV injected with BMH06-3Δ*tatD960*Δ*tatD825*. The log-rank test was used to compute *p*-values. A significant attenuation of pathogenicity was observed for the *tatD*-deficient strains compared to the wild-type strain, and increased survival was associated with deficiencies *in the tatD genes* (Figure 7A). No change was observed after 72 h (data not shown).

The bacterial load in the spleen after inoculating mice with *T. pyogenes* is shown in Figure 7B. Mice became depressed and exhibited edema of the eyelids 24 h after infection, and their food and water intake were reduced (Supplementary Table 3). The bacterial load in the spleen of mice inoculated with the BMH06-3Δ*tatD960* mutant or BMH06-3Δ*tatD825* mutant was not significantly changed compared with animals inoculated with the wild-type strain. However, the bacterial load in the spleen of mice inoculated with BMH06-3Δ*tatD960*Δ*tatD825* was significantly lower than that in the spleen of the mice from the other three groups. Based on these data, *tatD* gene deficiencies in *T. pyogenes* compromised virulence, and deletion strains lacking both the *tatD960* and *tatD825* genes exhibited significantly reduced infectivity in mice.

## Discussion

*T. pyogenes* synthesizes a variety of proteases and nucleases that are presumed to be associated with bacterial survival and pathogenicity (Rzewuska et al., 2019). TatD DNase is an evolutionarily conserved protein in bacteria, and our results indicate that *T. pyogenes* expresses two TatD DNases, TatD DNase 960, and TatD DNase 825. Both TpTatDs are Mg^2+^-dependent DNA endonucleases. TpTatDs promote biofilm formation and increase the virulence of *T. pyogenes* in mice.

Sequence characterization indicated that TatD DNases are evolutionarily conserved in bacteria, suggesting that TatD DNases may have similar structures and functions among bacteria (Figure 2D). The TatD protein was first identified to be encoded in the Tat operon in *E. coli* and is proposed to be a DNase associated with the Tat-associated quality control system (Wexler et al., 2000; Matos et al., 2009). We observed that TatD 960 and TatD 825 of *T. pyogenes* are both divalent metal ion-dependent DNases. They have similar functions to the TatD DNase of *E. coli*, *S*. *pneumoniae* and *Plasmodium knowlesi* (Figure 3A; Wexler et al., 2000; Jhelum et al., 2018; Zhou et al., 2018; Lee et al., 2020). Conversely, in *P. falciparum*, the activity of TatD DNase is repressed by divalent metal ions. This phenomenon may be caused by differences in the amino acid residues associated with the binding of metal ions by TatD DNases or by mutations in the TIM barrel structure to perform a specific role.

Some microorganisms can counteract NETs by secreting TatD DNases (Chang et al., 2016; Jhelum et al., 2018; Zhang et al., 2021). However, we found that TpTatDs were detected only intracellularly (Supplementary Figure 6). The *tatD* genes are probably involved in regulating other physiological activities in *T. pyogenes* rather than escaping neutrophil immunity. Deficiencies in TatD DNases decreased biofilm formation and the level of eDNA (Figures 5, 6A). eDNA has been confirmed to be one of the components of biofilms formed by numerous bacteria (Vorkapic et al., 2016; Schlafer et al., 2017). Autolysis is the mechanism by which eDNA is released from bacteria and is similar to apoptosis in eukaryotic cells (Montanaro et al., 2011; Vorkapic et al., 2016). Our results suggest that *tatD* deficiency might affect *T. pyogenes* biofilm formation through a reduction in bacterial cell lysis and the amount of eDNA present in the biofilm (Figures 5A, 6). The extracellular nuclease Nuc in *S. aureus* mediates the exodus of biofilm cells that is required for the development of the biofilm structure (Moormeier et al., 2014; Sultan et al., 2019). Unlike Nuc, TatD DNase tends to be involved intracellularly in promoting biofilm formation.

In our study, *tatD* deficiencies resulted in a lower bacterial burden in the spleen and compromised virulence of *T. pyogenes* in mice (Figure 7). Based on this result, TatD DNases are virulence factors in *T. pyogenes*, and the simultaneous presence of two DNases is necessary for full virulence. Similarly, the virulence and pathogenicity of *P. falciparum-* or *S. pneumoniae-*deficient TatD were also reduced compared to those of the wild-type strains (Chang et al., 2016; Jhelum et al., 2018). However, TatD in *P. falciparum* or *S. pneumoniae* functions as a virulence factor assisting pathogens in escaping NET capture. Meanwhile, TatD DNases from *T. pyogenes* existed in microbial cells and were not involved in escaping NET capture. Further studies are needed to determine whether TatD DNase is released from *T. pyogenes* via extracellular vesicles and participates in escaping NET capture under specific circumstances.

In conclusion, we obtained the following results: (i) TatD DNase 960 and TatD DNase 825 of *T. pyogenes* are DNA endonucleases with metal ion-dependent properties; (ii) TatD DNase tends to intracellularly influence biofilm formation; and (iii) the expression of TatD DNases is associated with virulence in *T. pyogenes*. This study deepens our understanding of the roles of DNases in *T. pyogenes*.

## Data Availability Statement

The original contributions presented in the study are included in the article/Supplementary Material, further inquiries can be directed to the corresponding authors.

## Ethics Statement

The animal study was reviewed and approved by the Ethical Committee of Shenyang Agricultural University, China.

## Author Contributions

ML, DZ, and ZZ conceived, wrote, reviewed, edited the manuscript, and designed the experiments. ZZ, YL, MC, YG, ZK, and CQ performed the experiments. YL and CT analyzed the data. LY, CT, and YG contributed reagents, materials, and analysis tools. All authors read and approved the final manuscript.

## Conflict of Interest

The authors declare that the research was conducted in the absence of any commercial or financial relationships that could be construed as a potential conflict of interest.

## Publisher’s Note

All claims expressed in this article are solely those of the authors and do not necessarily represent those of their affiliated organizations, or those of the publisher, the editors and the reviewers. Any product that may be evaluated in this article, or claim that may be made by its manufacturer, is not guaranteed or endorsed by the publisher.

## Footnotes

1
https://web.expasy.org/protparam/

2
http://pfam.xfam.org/

3
http://www.cbs.dtu.dk/services/SignalP-4.1/

4
https://www.genome.jp/tools-bin/clustalw

5
http://espript.ibcp.fr/ESPript/cgi-bin/ESPript.cgi

6
https://swissmodel.expasy.org/interactive

7
http://www.sbg.bio.ic.ac.uk/3dligandsite/

8
https://itol.embl.de/
