# Supplementary material for: TatD DNases Contribute to Biofilm Formation and Virulence in Trueperella pyogenes
Source: Front Microbiol. 2021 Nov 15;12:758465. doi: 10.3389/fmicb.2021.758465 (PMC8634637; doi:10.3389/fmicb.2021.758465)
Supplement: Supplementary file 1 [file Data_Sheet_1.docx]

Supplementary Material

# Supplementary Figures and Tables

## Supplementary Figures


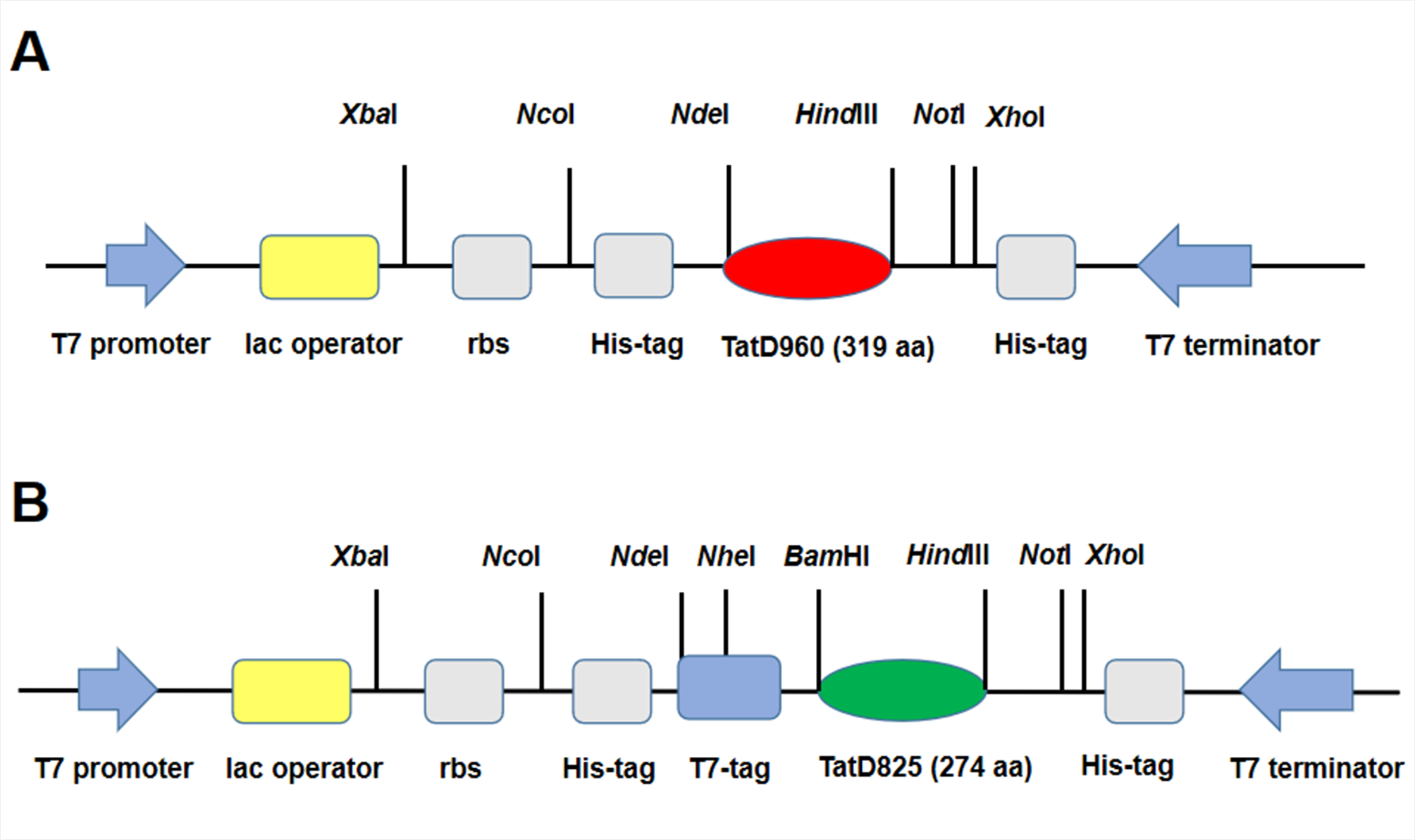


**Supplementary Figure S1. Diagram of expression cassettes of His-tagged recombination proteins.**

**(A)** TatD960 was ligated into the His-tagged protein expression cassette of pET28a from the enzymatic cleavage sites *Ned*I and *Hind*III. **(B)** TatD825 was ligated into the His-tagged protein expression cassette of pET28a from the enzymatic cleavage sites *Bam*HI and *Hind*III.


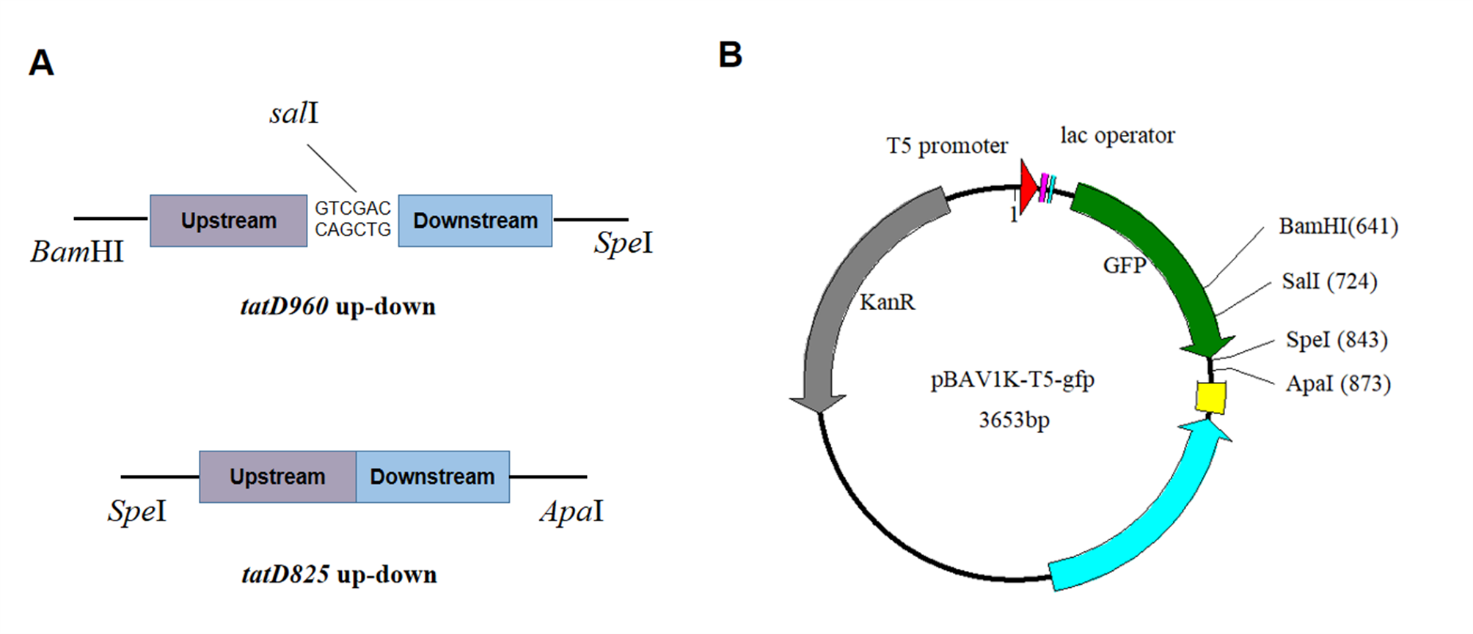


**Supplementary Figure S2. Construction of recombinant plasmids used for markerless gene deletion strategy.** **(A)** Upstream and downstream of *tatD960* were joined by *Sal*I and were inserted between *BamH*I and *Spe*I. Upstream-downstream sequences of *tatD825* were amplified by overlapping PCR and were ligated between *Spe*I and *Apa*I. (**B**) Location of the enzyme cleavage sites used in the genes of *tatD* knockout assay in the plasmid pBAV1K-T5-gfp.


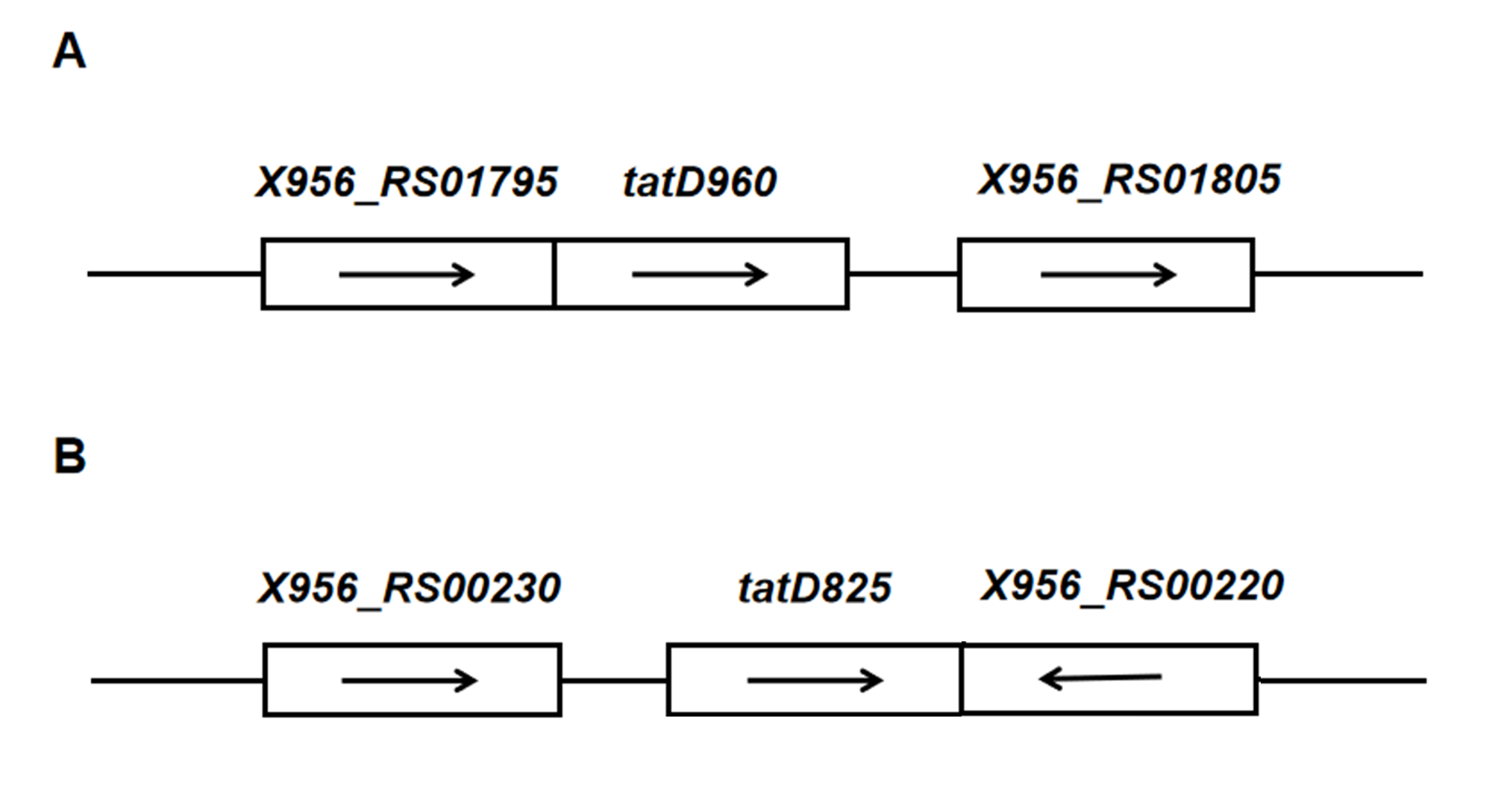


**Supplementary Figure S3. The location information of *tatD960* and *tatD825* in genomic DNA of *T. pyogenes.***

**(A)** *tatD960* is flanked by *X956_RS01795* (methionyl-tRNA synthetase, MetRS) and *X956_RS01805* (G5 domain-containing protein). **(B)** *tatD825* is flanked by *X956_RS00230* (thiamine biosynthesis protein, ThiF) and *X956_RS00220* (MFS transporter). Arrow in the rectangular box indicates the transcriptional orientation of the gene in the annotated TP8 genome (accession NO.: CP007003).


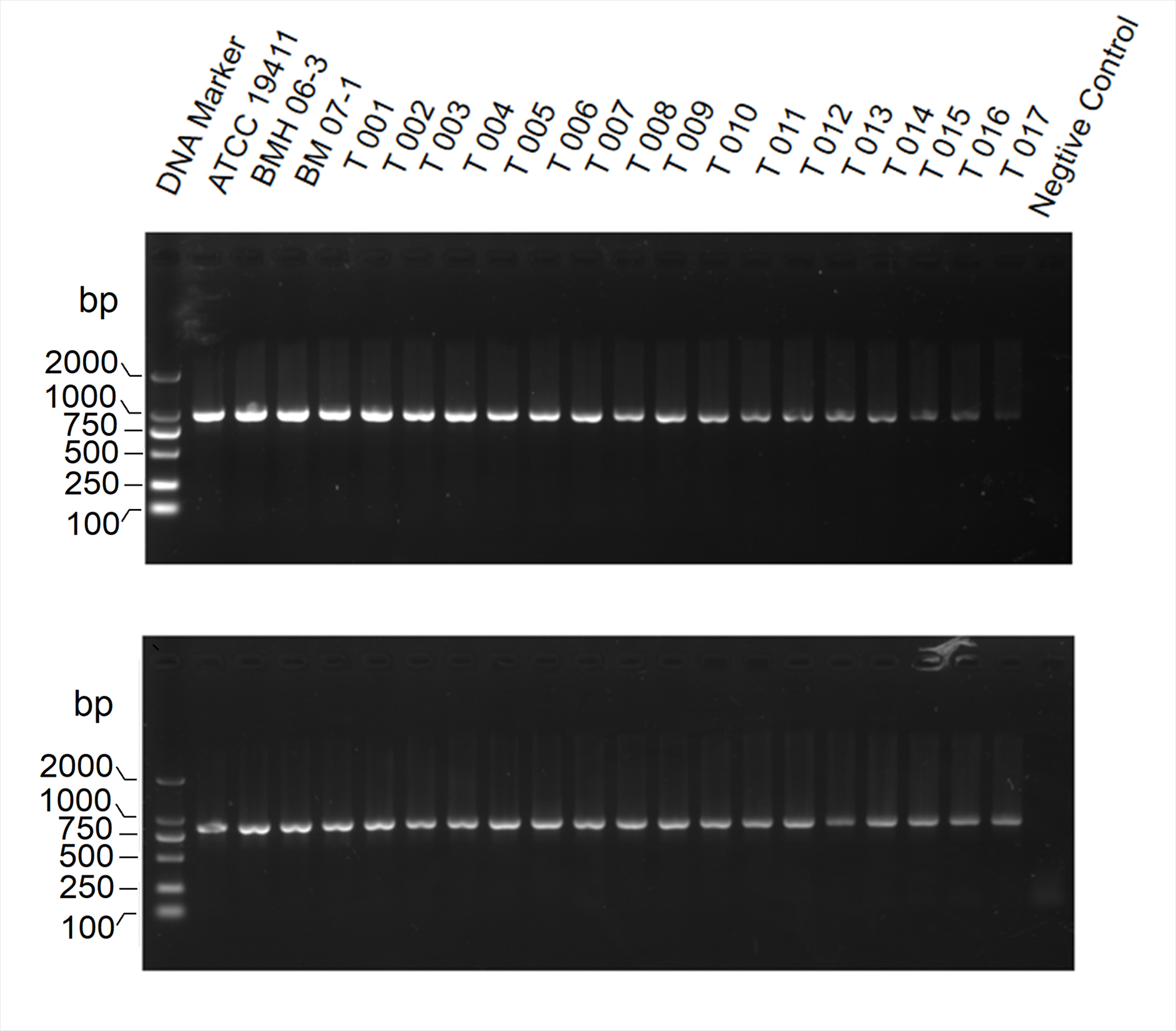


**Supplementary Figure S4. Results of screening for genes of *tatD* in 20 *T. pyogenes* isolates using agarose gel electrophoresis.** Top: *tatD960*, bottom: *tatD825*.


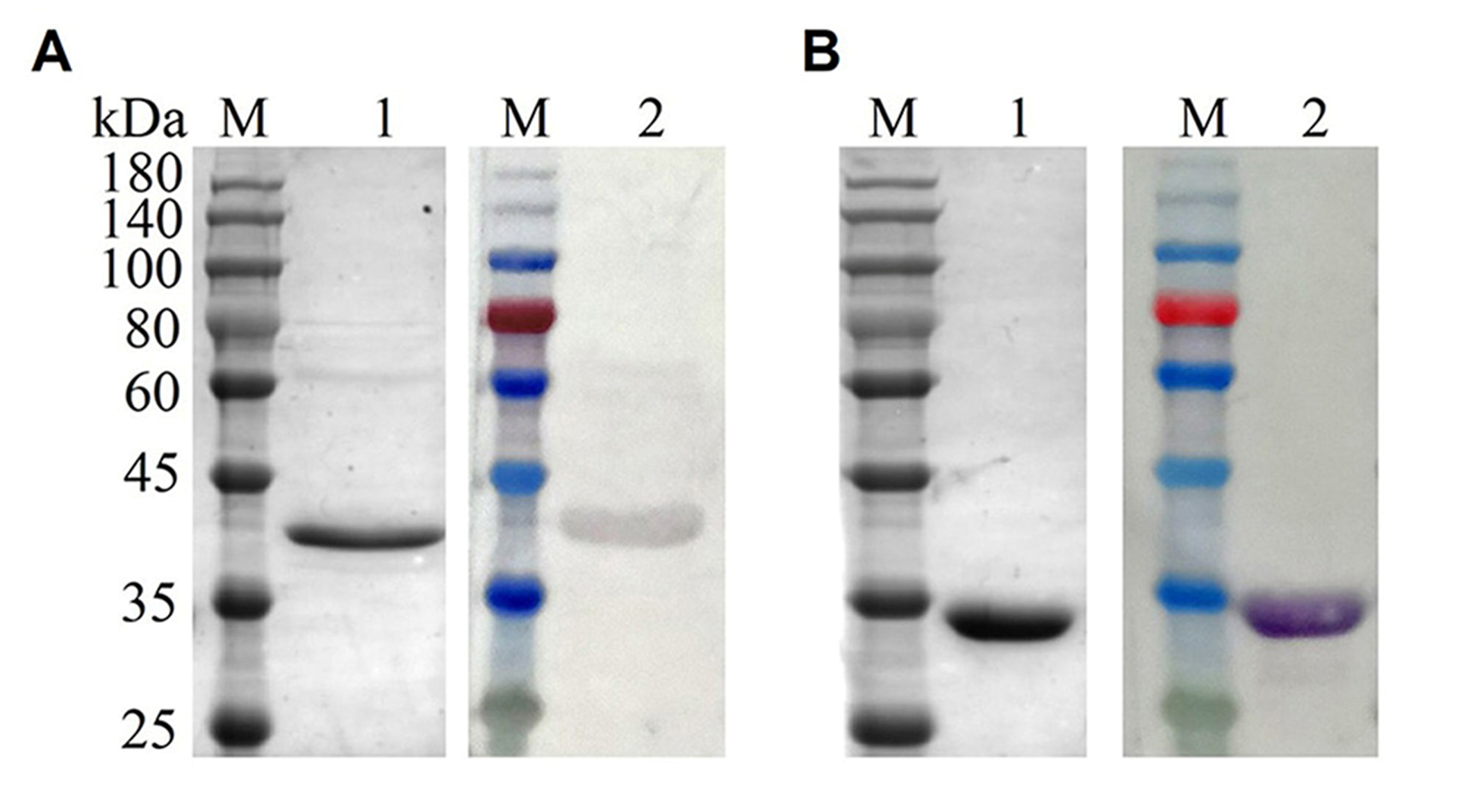


**Supplementary Figure S5. Purification of His-tagged TatD recombinant proteins and identification using Western blot. (A)** The purified His-tagged TatD 960 recombinant protein (37 kDa). **(B)** The purified His-tagged TatD 825 recombinant protein (32 kDa). Lane M: protein molecular weight standards, Lanes 1: SDS-PAGE analyses, Lanes 2: Western blot analyses.


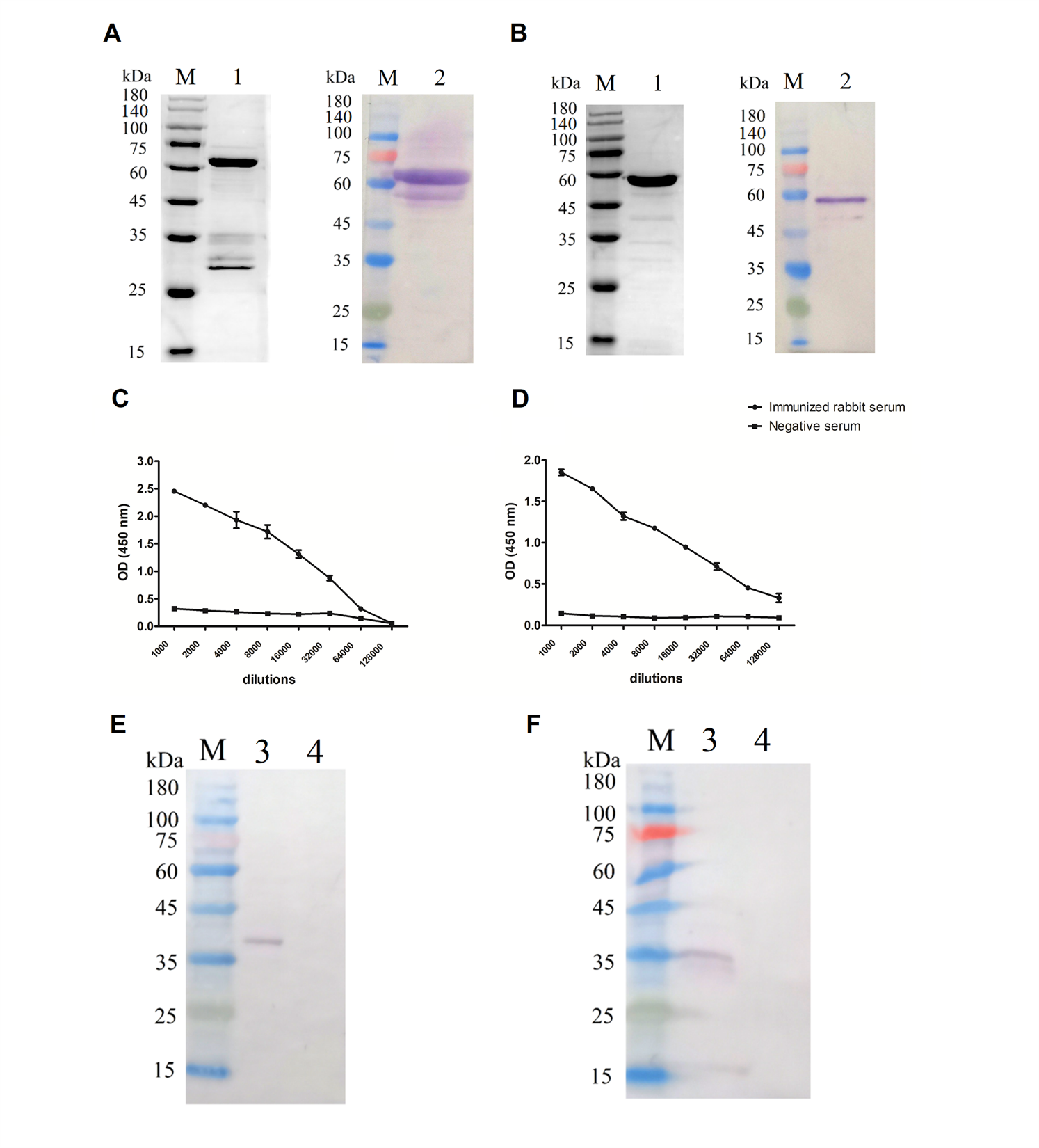


**Supplementary Figure S6. TatD DNases expressed in *T. pyogenes* were localized intracellularly.** **(A)** The purified GST-tagged TatD 960 recombinant protein (63 kDa). **(B)** The purified GST-tagged TatD 825 recombinant protein (58 kDa). Lane M: protein molecular weight standards, Lanes 1: SDS-PAGE analyses, Lanes 2: Western blot analyses. (**C**) The titer of anti-TpTatD960 in rabbit serum after fourth immunization. (**D**) The titer of anti-TpTatD825 in rabbit serum after fourth immunization. Indirect ELISA method for the detection of antibody titers in rabbit serum. Titers of the anti-TpTatD polyclonal antibodies were higher than 1:16,000 as defined by an OD ≥ 2.1 for the test sample/negative sample (P/N). **(E)** Detection of native TatD960 of *T. pyogenes* by Western blot **(F)** Detection of native TatD825 of *T. pyogenes* by Western blot. Lane 3, cell extract, Lane 4, culture supernatant, Lane M: protein molecular weight standards.

***
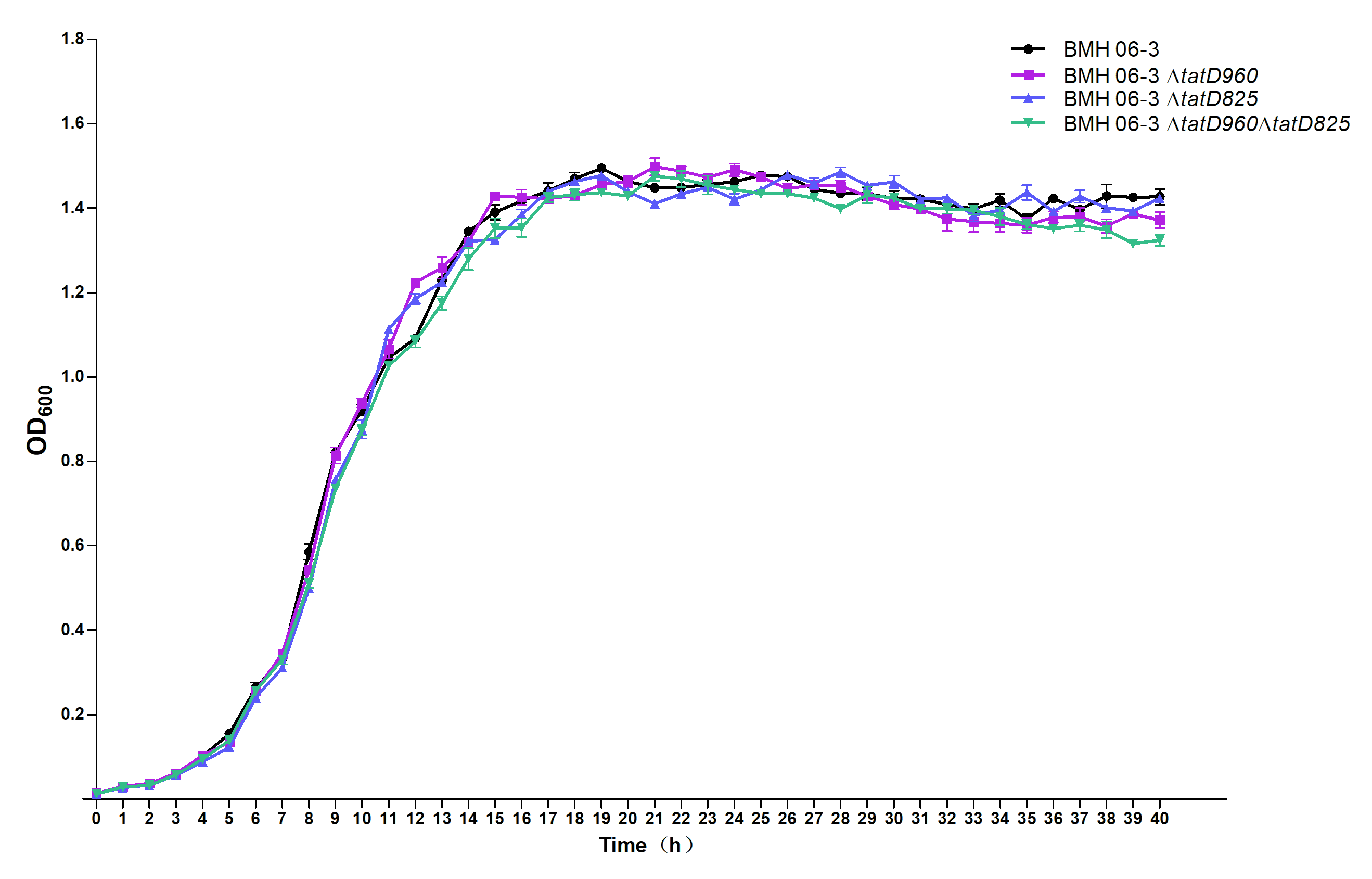
***

**Supplementary Figure S7. The growth curves of wild-type strain and *tatD* deficient mutants.** Data are presented as mean (± SD) of three replicates.

## Supplementary Tables

**Supplementary Table S1. Primer sequences for gene cloning, qPCR, and**

**gene knockout used in this study.**

| Primer name | Nucleotide sequence  (5'–3') | Product size (bp) |
| --- | --- | --- |
| *tatD960*-F | ATGGGTGATTCGACGTCGAAGA | 960 |
| *tatD960*-R | TCAGATGCCGTACACCTGCC |  |
| *tatD825*-F | ATGCTCCTTGACACCCACTT | 825 |
| *tatD825*-R | CTAATGAGAAAATGGTCGGCATGTGA |  |
| His-*tatD960*-F | CGCCATATGGGTGATTCGACGTCGAAGAAGGATC | 975 |
| His-*tatD960*-R | CCCAAGCTTTCAGATGCCGTACACCTGCC |  |
| His-*tatD825*-F | CGCGGATCCATGCTCCTTGACACCCACTTT | 843 |
| His-*tatD825*-R | GGGAAGCTTCTAATGAGAAAATGGTCGGCATGTGAC |  |
| GST-*tatD960*-F | CGCGGATCCATGGGTGATTCGACGTCGAAGAAG | 981 |
| GST-*tatD960*-R | TAAAGCGGCCGCTCAGATGCCGTACACCTG |  |
| GST-*tatD825*-F | CGGGATCCATGCTCCTTGACACCCACTTT | 841 |
| GST-*tatD825*-R | CCCTCGAGCTAATGAGAAAATGGTCGGCATGTGAC |  |
| *tatD960* L-F | CGGGATCCGGCGGAACCGGAGCCGAC | 1016 |
| *tatD960* L-R | GACGTCGACGCGCGCGGTGCCCAATC |  |
| *tatD960* R-F | CGCGTCGACCTACTCAGTGATCCCCAAACGCTC | 947 |
| *tatD960* R-R | GGACTAGTGCGCTCTCCTATTACTTCATGGGC |  |
| *tatD825* L-F | GGACTAGTCCTCGCCCTGTGTGTCAG | 1022 |
| *tatD825* L-R | AGTGGACAGTGCAGGGGTCACGACTGGGTGGGAG |  |
| *tatD825* R-F | CACCCAGTCGTGACCCCTGCACTGTCCACTCCGC | 1023 |
| *tatD825* R-R | ATTGGGCCCTGATGAATGCGGTGGTCATGTGG |  |

**Supplementary Table S2.** **Sources and Genbank ID of TatD DNases**

| No. | Source | Genbank ID |
| --- | --- | --- |
| 1 | *Actinomyces viscosus* | WP_003787606.1 |
| 2 | *Schaalia meyeri* | WP_074632781.1 |
| 3 | *Trueperella pyogenes* | WP_115324411.1 |
|  | *Trueperella pyogenes* | WP_024964333.1 |
| 4 | *Actinopolyspora mortivallis* | WP_019853010.1 |
| 5 | *Bifidobacterium* | WP_012578620.1 |
| 6 | *Denitrobacterium detoxificans* | WP_074777169.1 |
| 7 | *Dietzia maris* | WP_119192018.1 |
| 8 | *Mycobacterium kansasii* | WP_183155091.1 |
| 9 | *Streptomyces albidoflavus* | WP_164298596.1 |
| 10 | *Nocardiopsis alba* | WP_017533309.1 |
| 11 | *Thermomonospora echinospora* | WP_103937688.1 |
| 12 | *Chryseobacterium indologenes* | WP_079242074.1 |
| 13 | *Sphingobacterium mizutaii* | WP_185216125.1 |
| 14 | *Deinococcus* | WP_011529301.1 |
| 15 | *Bacillus anthracis* | KFJ83764.1 |
| 16 | *Listeria monocytogenes* | WP_003728894.1 |
| 17 | *Staphylococcus aureus* | WP_000904310.1 |
| 18 | *Streptococcus pneumoniae* | WP_000575597.1 |
| 19 | *Clostridium paraputrificum* | WP_105338205.1 |
| 20 | *Blastopirellula marina* | WP_105338205.1 |
| 21 | *Azospirillum lipoferum* | WP_149235179.1 |
| 22 | *Rickettsia conorii* | WP_016926491.1 |
| 23 | *Cupriavidus basilensis* | WP_059412941.1 |
| 24 | *Hylemonella gracilis* | WP_051509890.1 |
| 25 | *Sutterella wadsworthensis* | WP_016475028.1 |
| 26 | *Rhodocyclus tenuis* | WP_153590069.1 |
| 27 | *Desulfohalobium retbaense* | WP_015751171.1 |
| 28 | *Desulfovibrio alaskensis* | WP_027182074.1 |
| 29 | *Chondromyces crocatus* | WP_050430087.1 |
| 30 | *Anaerobiospirillum succiniciproducens* | WP_027940046.1 |
| 31 | *Teredinibacter turnerae* | WP_018276642.1 |
| 32 | *Rheinheimera baltica* | WP_027672051.1 |
| 33 | *Escherichia coli* | BAP40087.1 |
| 34 | *Pectobacterium atrosepticum* | WP_207193783.1 |
| 35 | *Serratia marcescens* | WP_195315713.1 |
| 36 | *Methylococcus capsulatus* | WP_017366306.1 |
| 37 | *Cobetia marina* | WP_175088193.1 |
| 38 | *Acinetobacter radioresistens* | WP_119878378.1 |
| 39 | *Vibrio splendidus* | WP_114633967.1 |
| 40 | *Xanthomonas campestris* | WP_166751037.1 |
| 41 | *Hydrogenophilus thermoluteolus* | WP_119335103.1 |
| 42 | *Leptonema illini* | WP_040918848.1 |
| 43 | *Treponema denticola* | WP_147624127.1 |
| 44 | *Fervidobacterium islandicum* | WP_033190862.1 |
| 45 | *Plasmodium falciparum* Protists | PKC48074.1 |
| 46 | *Plasmodium knowlesi* Protists | SBO28267.1 |
| 47 | *Entamoeba histolytica* Protists | XP_651470.1 |
| 48 | *Entamoeba nuttalli* Protists | XP_008857182.1 |
| 49 | *Thecamonas trahens* Protists | XP_013757624.1 |
| 50 | *Trypanosoma brucei* Protists | XP_828684.1 |
| 51 | *Aureobasidium melanogenum* FUNGI | XP_040879870.1 |
| 52 | *Sphaerulina musiva* FUNGI | XP_016756671.1 |
| 53 | *Paraphaeosphaeria sporulosa* FUNGI | XP_018030096.1 |
| 54 | *Macroventuria anomochaeta* FUNGI | XP_033566227.1 |
| 55 | *Aspergillus fumigatus* FUNGI | XP_752125.1 |
| 56 | *Penicillium digitatum* FUNGI | XP_014537925.1 |
| 57 | *Rasamsonia emersonii* FUNGI | XP_013324933.1 |
| 58 | *Talaromyces marneffei* FUNGI | XP_002144407.1 |
| 59 | *Talaromyces stipitatus* FUNGI | XP_002341145.1 |
| 60 | *Coccidioides immitis* FUNGI | XP_001244697.2 |
| 61 | *Coccidioides posadasii* FUNGI | XP_003071337.1 |
| 62 | *Colletotrichum orchidophilum* FUNGI | XP_022469794.1 |
| 63 | *Colletotrichum scovillei* FUNGI | XP_035328892.1 |
| 64 | *Colletotrichum karsti* FUNGI | XP_038739949.1 |
| 65 | *Sodiomyces alkalinus* FUNGI | XP_028471302.1 |
| 66 | *Metarhizium acridum* FUNGI | XP_007811941.1 |
| 67 | *Pochonia chlamydosporia* FUNGI | XP_018149186.1 |
| 68 | *Beauveria bassiana* FUNGI | XP_008598313.1 |
| 69 | *Cordyceps militaris* FUNGI | XP_006667082.1 |
| 70 | *Phaeoacremonium minimum* FUNGI | XP_007918921.1 |
| 71 | *Babesia bigemina* Protists | XP_012766110.1 |
| 72 | *Babesia bovis* Protists | XP_001610292.1 |
| 73 | *Theileria equi* Protists | XP_004829323.1 |
| 74 | *Cryptosporidium muris* Protists | XP_002140135.1 |
| 75 | *Eimeria acervulina* Protists | XP_013247879.1 |
| 76 | *Eimeria maxima* Protists | XP_013336952.1 |
| 77 | *Eimeria mitis* Protists | XP_013352294.1 |
| 78 | *Eimeria necatrix* Protists | XP_013433460.1 |
| 79 | *Eimeria tenella* Protists | XP_013229677.1 |
| 80 | *Toxoplasma gondii* Protists | XP_018638195.1 |
| 81 | *Gregarina niphandrodes* Protists | XP_011133136.1 |
| 82 | *Tetrahymena thermophila* Protists | XP_001017495.2 |
| 83 | *Phytophthora ramorum* Protists | AIF71166.1 |
| 84 | *Phytophthora sojae* Protists | AIF71150.1 |
| 85 | *Aureococcus anophagefferens* Protists | XP_009034472.1 |
| 86 | *Chondromyces crocatus* | WP_050430087.1 |
| 87 | *Anaerobiospirillum succiniciproducens* | WP_027940046.1 |
| 88 | *Teredinibacter turnerae* | WP_018276642.1 |

**Supplementary Table S3. Clinical symptom for infected mice**

| Group (Injected) | Feeding intake  per mouse (g/d) | Water intake  per mouse(mL/d) | Eye discharge |
| --- | --- | --- | --- |
| BMH06-3 | 1.8 | 4.0 | Pyogenic |
| BMH06-3△*tatD960* | 2.0 | 4.3 | Pyogenic |
| BMH06-3△*tatD825* | 2.7 | 4.0 | Pyogenic |
| BMH06-3△*tatD960*△*tatD825* | 2.3 | 4.7 | Pyogenic |
| 0.9% saline | 5.5 | 8.3 | None |
